# Minimally-Invasive Surgical Approach to Congenital Dacryostenosis: Proposal for a New Protocol

**DOI:** 10.3389/fped.2021.569262

**Published:** 2021-02-15

**Authors:** Stefano Pensiero, Laura Diplotti, Gianluca Visalli, Luca Ronfani, Manuela Giangreco, Egidio Barbi

**Affiliations:** ^1^Department of Ophthalmology, Institute for Maternal and Child Health - IRCCS Burlo Garofolo, Trieste, Italy; ^2^Department of Medical, Surgical and Health Sciences, University of Trieste, Trieste, Italy; ^3^Clinical Epidemiology and Public Health Research Unit, Institute for Maternal and Child Health - IRCCS Burlo Garofolo, Trieste, Italy; ^4^Department of Pediatrics, Institute for Maternal and Child Health - IRCCS Burlo Garofolo, Trieste, Italy

**Keywords:** congenital dacryostenosis, congenital nasolacrimal duct obstruction, lacrimal drainage system probing, silicone dacryointubation, minimally-invasive surgery, timing of surgery, protocol, children

## Abstract

**Background:** Congenital dacryostenosis is one of the most common ophthalmological disorders in infants, with a high spontaneous resolution rate. In patients unresponsive to conservative treatment, the first-line approach is lacrimal drainage system probing, thought there is no clear consensus on optimal timing of surgery. The optimal treatment of patients unresponsive to primary probing is also controversial.

**Objectives:** The aim of this study is to assess the optimal timing of probing in children with congenital dacryostenosis. Other purposes are to evaluate the efficacy of repeated probing and dacryointubation in patients unresponsive to the initial surgery without evident lacrimal outflow dysgenesis, and to determine the epidemiology of these maldevelopments.

**Methods:** A retrospective consecutive cohort study was conducted in 625 eyes of 457 patients aged 7–48 months who underwent surgery for dacryostenosis. Patients were divided into 4 cohorts according to the timing of surgery. Data were analyzed using Fisher's test.

**Results:** The success rate of primary probing was high, without significant differences between cohorts. One-third of recurrences were related to maldevelopments, the other two-thirds were treated with a second probing or dacryointubation, with high success rates, that did not significantly differ between the procedures. All cases unresponsive to the second surgery were resolved with dacryointubation.

**Conclusions:** Probing is highly effective and its outcome is not affected by timing of surgery. Nevertheless, we advocate for early intervention, in order to identify possible maldevelopments, which require more invasive management. In patients unresponsive to primary probing, without evident maldevelopments, repeated probing should still be considered as the first-line approach, since it's less invasive but similarly effective to dacryointubation.

## Introduction

Congenital dacryostenosis (congenital nasolacrimal duct obstruction or CNLDO) is an inborn blockage of the lacrimal drainage structures. It usually caused by the incomplete canalization of the nasolacrimal duct at the Hasner valve level (1), and less frequently due to lacrimal outflow dysgenesis (LOD), i.e., bone abnormalities, membranes and/or stenosis at any part of the lacrimal drainage system (2, 3); either sporadic or associated with systemic syndromes and craniofacial abnormalities (4, 5).

CNLDO is the leading cause of persistent epiphora and ocular discharge in the pediatric population, affecting up to 20% of infants (6). It is estimated to resolve spontaneously or in response to conservative management (i.e., effective Crigler lacrimal sac massage and topical antibiotic therapy) within the first year of life in almost 90% of cases (7, 8). Therefore, surgical approach is only required in patients unresponsive to conservative treatment or presenting with chronic or recurrent bacterial conjunctivitis, in order to prevent dacryocystitis (9). In such cases the standard of care is lacrimal drainage system probing and irrigation, under local or general anesthesia, even though there is no clear consensus on optimal timing of surgery.

The overall success rate of primary probing in CNLDO (75–89%) is high (6, 10, 11). Cases unresponsive to the initial surgery may benefit from second probing, dacryointubation, balloon catheter dilation, dacryocystorhinostomy (DCR) or a combination of procedures (12), with variable outcomes.

The aim of this study is to assess the optimal timing of surgery in a pediatric population with CNLDO, by evaluating the success rate of primary probing among various age groups. Other purposes are to evaluate the efficacy of repeated probing and of silicone dacryointubation in patients unresponsive to the initial probing with no evident LOD, and to determine the epidemiology of these maldevelopments in the studied population.

## Materials and Methods

In this retrospective consecutive cohort study, medical records of all patients aged 7–48 months who underwent primary probing for CNLDO at the Ophthalmology Department of the Institute for Maternal and Child Health of Trieste—*IRCCS Burlo Garofolo* (Italy) between 2008 and 2018, were investigated. Patients with coexistent systemic syndromes and craniofacial abnormalities were excluded. The research was approved by the Institutional Review Board of *IRCCS Burlo Garofolo* and adheres to the tenets of the Declaration of Helsinki.

**Figure 1:**
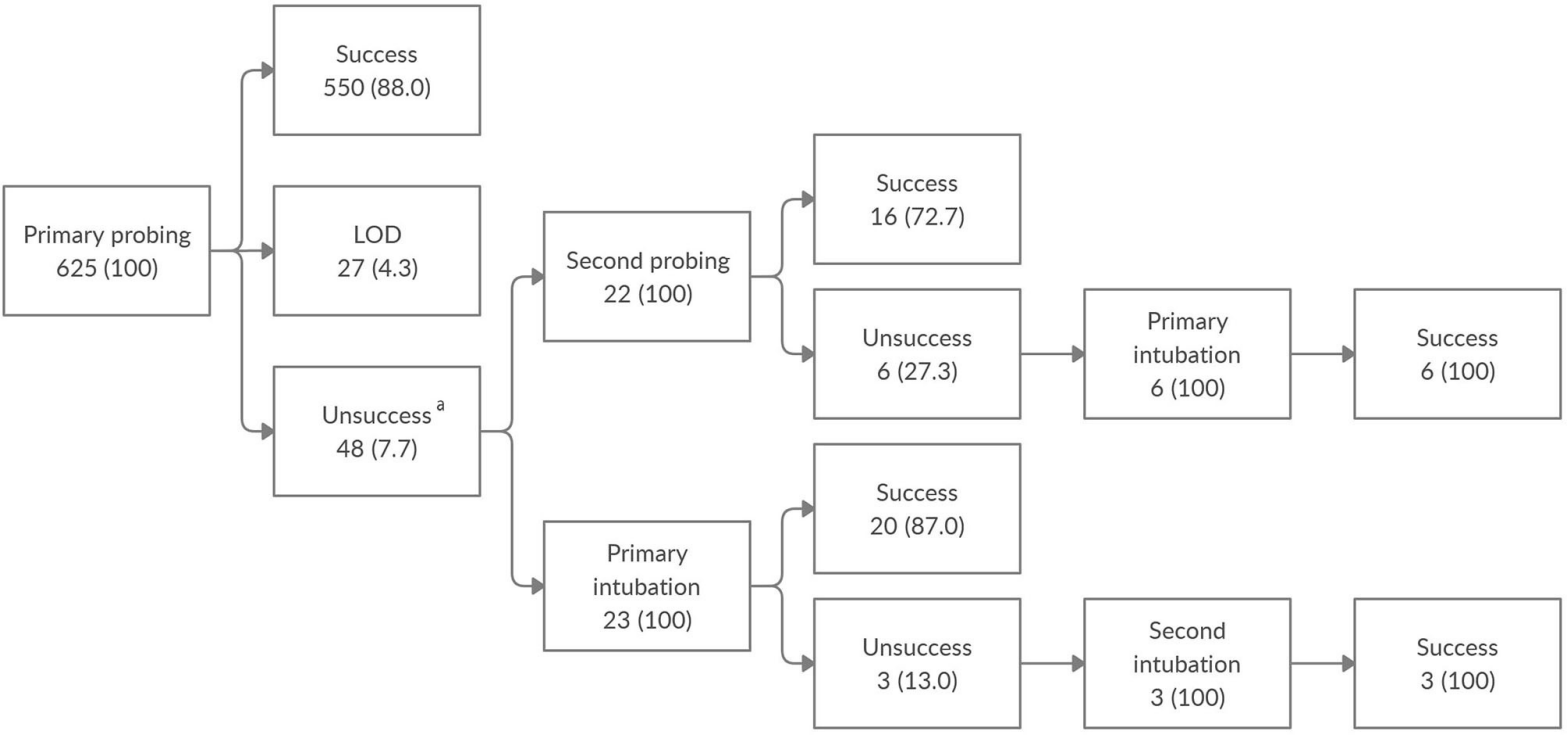
Clinical outcome of nasolacrimal duct obstruction surgery among the enrolled population. Data are expressed as number of eyes (%). LOD, lacrimal outflow dysgenesis. ^a^Out of 48 eyes unresponsive to the initial probing, without LOD, 3 were lost to follow-up.

All naïve patients underwent lacrimal drainage system probing and irrigation. Patients in whom the initial surgery failed and LOD was not found underwent a second probing or a bicanalicular silicone intubation. All patients in whom the second surgery failed underwent dacryointubation (Figure 1). In patients unresponsive to surgical treatment who underwent several operations, the least time interval between two consecutive procedures was 6 months. In patients who underwent dacryointubation, tube removal was performed 2 months after surgery.

All procedures were performed under general anesthesia, using intravenous Propofol, in the presence of an anaesthesiologist, with different ventilation modalities according to the type and duration of intervention. Lacrimal drainage system probing took about 5–10 min and was performed in spontaneous ventilation; whereas, silicone intubation took about 15–20 min and was performed in assisted ventilation with laryngeal mask. Sometimes, intravenous cannulation was performed after inhalational of Sevoflurane. All the surgical procedures were performed with the same technique, by the 3 ophthalmologists working at the lacrimal surgery service.

Post-operatively patients received topical steroid and antibiotic combination 4 times per day for a week.

In patients with bilateral CNLDO, both surgeries were performed at the same time and both eyes were included in this study.

Clinical data collected included: gender, age at the time of surgery (patients were divided into 4 age groups: 7–12, 13–24, 25–36, and 37–48 months), type of surgical procedure (lacrimal drainage system probing or intubation), surgical outcome at 6 months (resolution or persistence of signs), and presence of LOD (defined as the inability to advance the probe in the lacrimal drainage system).

Descriptive statistics were used to report the results. Categorical variables were presented as numbers and percentages, continuous variables as mean value with standard deviation. The Fisher's exact test was used to assess for statistical significance, which was defined as a *P*-value (*P*) < 0.05. All analyses were conducted using SAS software, Version 9.4 (SAS Institute Inc., Cary, NC, USA).

## Results

Overall, 625 eyes of 457 patients, 255 (55.8%) males and 202 (44.2%) females, were enrolled in this study. CNLDO was unilateral in 289 (63.2%) patients and bilateral in 168 (36.8%). At the time of the first surgical operation the mean age was 21.4 ± 9 months: 52 (11.4%) patients underwent primary probing at age 7–12 months, 267 (58.4%) at 13–24 months, 100 (21.9%) at 25–36 months, and 38 (8.3%) at 37–48 months (Table 1). The outcomes of CNLDO surgery among the enrolled population divided into 4 age groups are shown in Figure 1 and Table 2.

**Table 1:** Demographic characteristics of the enrolled population.

**Age group**	**7–12 Months**	**13–24 Months**	**25–36 Months**	**37–48 Months**	**Overall**
Patients	52 (11.4)	267 (58.4)	100 (21.9)	38 (8.3)	457 (100)
Males	26 (5.7)	148 (32.4)	60 (13.1)	21 (4.6)	255 (55.8)
Females	26 (5.7)	119 (26.0)	40 (8.8)	17 (3.7)	202 (44.2)
OD	16 (3.5)	93 (20.4)	36 (7.9)	10 (2.2)	155 (33.9)
OS	17 (3.7)	85 (18.6)	18 (3.9)	14 (3.1)	134 (29.3)
OU	19 (4.2)	89 (19.5)	46 (10.1)	14 (3.1)	168 (36.8)

*Results are expressed as number of patients (%). OD, oculus dexter (right eye); OS, oculus sinister (left eye); OU, oculus uterque (both eyes)*.

**Table 2:** Success rate of nasolacrimal duct obstruction surgery among the enrolled population divided into 4 age groups.

	**7–12 Months**	**13–24 Months**	**25–36 Months**	**37–48 Months**	**Overall**
**First surgery**					
Primary probing	71 (100)	356 (100)	146 (100)	52 (100)	625 (100)
▪ Success	57 (80.3)	313 (87.9)	136 (93.2)	44 (84.6)	550 (88.0)
▪ Unsuccess	9 (12.7)	30 (8.4)	6 (4.1)	3 (5.8)	48 (7.7)
▪ LOD	5 (7.0)	13 (3.7)	4 (2.7)	5 (9.6)	27 (4.3)
**Second surgery**^**a**^					
Second probing	-	6 (100)	11 (100)	5 (100)	22 (100)
▪ Success	-	5 (83.3)	6 (54.5)	5 (100)	16 (72.7)
▪ Unsuccess	-	1 (16.7)	5 (45.5)	0 (0.0)	6 (27.3)
Primary intubation	-	8 (100)	7 (100)	8 (100)	23 (100)
▪ Success	-	7 (87.5)	6 (85.7)	7 (87.5)	20 (87.0)
▪ Unsuccess	-	1 (12.5)	1 (14.3)	1 (12.5)	3 (13.0)
**Third surgery**					
Primary intubation	-	-	2 (100)	4 (100)	6 (100)
▪ Success	-	-	2 (100)	4 (100)	6 (100)
▪ Unsuccess	-	-	0 (0)	0 (0)	0 (0)
Second intubation	-	-	2 (100)	1 (100)	3(100)
▪ Success	-	-	2 (100)	1 (100)	3(100)
▪ Unsuccess	-	-	0 (0)	0 (0)	0 (0)

*Results are expressed as number of eyes (%). LOD, lacrimal outflow dysgenesis*.

a *Out of 48 eyes unresponsive to the initial probing, with no LOD, 3 were lost to follow-up*.

The overall success rate of primary probing was 88.0%: 80.3% in the 7–12 months group, 87.9% in the 13–24 months group, 93.2% in the 25–36 months group, and 84.6% in the 37–48 months group.

Out of 75 cases of probing failure, 27 (36%) eyes were diagnosed with congenital LOD, 3 (4%) were lost to follow up and the remaining 45 (60%) were treated with a second probing or a dacryointubation. No patients underwent the second surgery before age 13 months.

No statistically significant difference was found between success rate of the initial probing in the 4 age groups (*P* = 0.11).

The overall success rate of second probing was 72.7%: 83.3% in the 13–24 months group, 54.5% in the 25–36 months group, and 100% in the 37–48 months group.

The overall success rate of primary intubation was 87.0%: 87.5% in the 13–24 months group and in the 37–48 months group, 85.7% in the 25–36 months group.

As the number of cases who underwent second probing and primary intubation were similar (22 and 23 eyes, respectively) we compared the success rates of these procedures. No statistically significant difference was found between the outcome of second probing and of primary intubation (*P* = 0.28).

All cases unresponsive to the second surgery were treated with dacryointubation, with a 100% success rate. No patients underwent the third surgery before age 25 months.

The overall incidence of congenital LOD was 4.3%. Proximal lacrimal outflow tight stenosis and atresia affected 9 (33.3%) and 2 (7.4%) eyes, respectively, whereas distal tight stenosis and atresia affected 12 (44.5%) and 4 (14.8%) eyes, respectively. The demographic characteristics of children with LOD are depicted Table 3.

**Table 3:** Congenital lacrimal outflow dysgenesis among the enrolled population divided into 4 age groups.

**Age group**	**7–12 Months**	**13–24 Months**	**25–36 Months**	**37–48 Months**	**Overall**
Patients	3 (16.7)	10 (55.6)	2 (11.1)	3 (16.7)	18 (100)
Males	2 (11.1)	3 (16.7)	1 (5.6)	1 (5.6)	7 (38.9)
Females	1 (5.6)	7 (38.9)	1 (5.6)	2 (11.1)	11 (61.1)
OD	1 (5.6)	6 (33.3)	-	-	7 (38.9)
OS	-	1 (5.6)	-	1 (5.6)	2 (11.1)
OU	2 (11.1)	3 (16.7)	2 (11.1)	2 (11.1)	9 (50.0)

*Results are expressed as number of patients (%). OD, oculus dexter (right eye); OS, oculus sinister (left eye); OU, oculus uterque (both eyes)*.

## Discussion

Congenital dacryostenosis is one of the most common ophthalmological disorders in infants. It usually resolves spontaneously or in response to effective lacrimal sac massage within the first year of life (8). Therefore, surgical approach is only required in patients with persistent signs, unresponsive to conservative treatment. In such cases the standard of care is lacrimal drainage system probing, even though debate continues about optimal timing of surgery (9, 13).

In this study most patients (58.4%) underwent surgery between 13 and 24 months of age, in accordance with the previous indications based on the data of Nelson et al. (14), who observed that probing is seldom required before the first year of life, as, in the majority of cases CNLDO resolves by age 13 months.

The overall success rate of primary probing in our study was 88.0%, ranging from 80.3% in patients aged 7–12 months to 93.2% in patients aged 25–36 months, without statistically significant difference between rates in the 4 age groups. Similar efficacy data (75–89%) were reported in the literature (6, 10, 11), though, there is no consensus on the correlation between age at the time of surgery and surgical outcome. Some authors assert that younger patients undergoing probing have the better prognosis (8, 13, 15), others found that aging has no effect on the success rate of initial probing (16–20). According to Robb, probing failure rate seems to be related to the presence of nasolacrimal duct anatomy anomalies rather than to a delay in probing (21). Our data suggest similar conclusions: we found the higher success rate in patients with incomplete canalization of the nasolacrimal duct at Hasner valve level, with one-third of recurrences occurring in patients with LOD. Even surgeons' experience may play a role in surgical outcome. As CNLDO surgery under general anesthesia requires less technical skills than under local anesthesia, all procedures in this study were performed under general anesthesia, in order to minimize failure related to surgical execution.

In 2016, the US Food and Drug Administration published a warning regarding the prolonged (>3 h) or repeated exposure to anesthetics in children younger than 3 years, as it may affect brain development (22, 23). This concern was mainly based on animal studies, whereas clinical studies seem ambivalent (24–26). Emerging clinical studies support a dose-response relationship between general anesthesia exposure and neurotoxicity (24), suggesting that, in children, a single exposure (24) and/or exposures up to 1 h (25) are not associated with detectable risks of long-term consequences. In our cohort, surgery never lasted longer than 20 min, and patients needing reintervention underwent at most 3 consecutive operations, the least time interval between two consecutive procedures being 6 months. Hence, in our opinion, patients' exposure to anesthesia in our study was minimal, and the potential risk for neurotoxicity negligible.

The optimal treatment of patients unresponsive to primary probing is also controversial. Options include second probing, intubation, balloon catheter dilation, DCR or a combination of procedures (12), with variable success rates.

In this study, after primary probing failure, out of 45 eyes without congenital LOD, 22 eyes underwent a second probing and 23 eyes a primary silicone dacryointubation, with a success rate of 72.7 and 87%, respectively. No statistically significant difference between the outcomes of these procedures was found. Our results are similar to the ones reported in the literature, as the resolution rates of second probing and dacryointubation are estimated to range from 52 to 100% and from 62 to 100%, respectively (6, 27–33). All cases unresponsive to the second surgery were resolved with dacryointubation. Hence, according to our study results, except for CNLDO related to LOD, all cases unresponsive to primary probing can still be resolved with a minimally-invasive surgical approach. Overall, CNLDO surgery requires a minimally-invasive anesthesiological approach, as it lasts <20 min, with an overall exposure <1 h in patients needing up to 3 operations. Of note, probing is similarly effective to silicone dacryointubation, yet it is technically easier to perform, requires less invasive anesthesia and only little postoperative compliance. Therefore, even though a limitation of this study is its retrospective observational nature, our findings suggest a 3-step operative protocol for the surgical approach to CNLDO. Patients with persistent CNLDO after a single failed probing and no evident LOD, should undergo a second probing, whereas intubation has to be considered as a third-line treatment.

LOD is a usually sporadic and isolated condition that may cause probing failure in patients with CNLDO (34, 35). In this study, out of 75 eyes unresponsive to primary probing 27 (36%) were found to have LOD (59.3% in its distal tract and 40.7% in its proximal tract), with an overall incidence of 4.3%. Similar results have been described in the literature, though the proximal tract is usually most frequently involved (34–36). CNLDOs related to LOD represent the more complex cases, as require more invasive management, i.e., DCR, and show less predictable prognosis (7, 37–40).

## Conclusions

Given the patients' very young age, the least invasive surgical and anesthesiological approach to persistent CNLDO should be used.

Our data suggest that, lacrimal drainage system probing should be considered as the standard of care in CNLDO treatment at any age, as it's highly effective and its outcome is not affected by timing of surgery. Nevertheless, we advocate for early intervention, as probing helps to early identify possible congenital LOD, which usually require more invasive management.

According to our study results, we believe that in patients unresponsive to primary probing, with no evident maldevelopments, a repetition of probing should still be considered as the first-line approach, before undergoing a silicone dacryointubation, since it is less invasive but similarly effective.

## Data Availability Statement

The original contributions presented in the study are included in this published article; further inquiries can be directed to the corresponding author LD.

## Ethics Statement

This research was approved by the Institutional Review Board of the Institute for Maternal and Child Health—*IRCCS Burlo Garofolo*, and adheres to the tenets of the Declaration of Helsinki. Proper informed consent for the treatment was obtained prospectively from the parents of all subjects who underwent CNLDO surgery.

## Author Contributions

SP and EB conceived and designed the study. GV acquired the data. LR and MG performed the statistical analysis. All the authors discussed the results and contributed to the interpretation of the data. SP and LD drafted the manuscript, the tables, and the figure. All the authors had full access to all the data in the study and take responsibility for the integrity of the data and the accuracy of the data analysis as well as the decision to submit for publication.

## Conflict of Interest

The authors declare that the research was conducted in the absence of any commercial or financial relationships that could be construed as a potential conflict of interest.

## Abbreviations

CNLDO: congenital nasolacrimal duct obstruction
LOD: lacrimal outflow dysgenesis
DCR: dacryocystorhinostomy
P: *P*-value
OD: oculus dexter (right eye)
OS: oculus sinister (left eye)
OU: oculus uterque (both eyes).
